# Statin prescribing patterns in cardiovascular risk management among outpatients with type 2 diabetes: Real-world practices at a Vietnamese general hospital

**DOI:** 10.1371/journal.pone.0343313

**Published:** 2026-02-27

**Authors:** Long Bui, Thu Thi Le, Nhung Hong Dao, Hoa Thi Nguyen, Thao Thi Nguyen, Van Thi Thuy Pham, Thao Thi Bich Cao, Phuong Thanh Nguyen, Phuong T. Xuan Dong

**Affiliations:** 1 Department of Interventional Cardiology, Friendship Hospital, Hai Ba Trung, Hanoi, Vietnam; 2 Faculty of Pharmacology and Clinical Pharmacy, Hanoi University of Pharmacy, Hoan Kiem, Hanoi, Vietnam; 3 Department of Pharmacy, Friendship Hospital, Hai Ba Trung, Hanoi, Vietnam; 4 Department of Internal Medicine A, Friendship Hospital, Hai Ba Trung, Hanoi, Vietnam; University of Perugia Department of Medicine: Universita degli Studi di Perugia Dipartimento di Medicina, ITALY

## Abstract

**Objective:**

This study aimed to evaluate the current prescribing patterns of statin therapy among outpatients with type 2 diabetes at Friendship Hospital, with a focus on alignment between treatment intensity with cardiovascular risk factors.

**Methods:**

A retrospective cohort study was conducted at Friendship Hospital from December 2022 to December 2023. Patient aged 40–75 years with type 2 diabetes mellitus who attended follow-up visits were included using stratified random sampling by treatment department. Clinical data were extracted from medical records to assess cardiovascular risk based on the 2023 American Diabetes Association guideline and to document statin use, intensity, treatment continuity and related factors. Multivariate logistic regression was performed to identify factors associated with whether or not patients were prescribed statins.

**Results:**

Among 407 patients, 30.5% were classified as having established ASCVD, 55.6% as having high cardiovascular risk, and 99.8% of patients were eligible for indication of high intensity statin. Despite this, none received high-intensity statin. Most patients (77.4%) were prescribed moderate-intensity statins, 8.6% received low-intensity statins, and 14.0% were not prescribed any statin. A total of 58.5% of patients received uninterrupted statin prescriptions over one year, whereas 54.3% underwent changes in statin intensity during the follow-up period. Statin prescription (versus non-prescription) was significantly influenced by the treatment department, ASCVD status, and the presence of dyslipidemia (p < 0.05).

**Conclusion:**

Although statin use was common, the intensity and continuity of treatment were often inconsistent with cardiovascular risks. The absence of high-intensity statin prescriptions in a population largely at very high risk may limit the potential benefits of lipid-lowering therapy. These findings highlight the need to strengthen adherence to guideline-directed statin therapy in the management of type 2 diabetes mellitus.

## Introduction

Type 2 diabetes mellitus (T2DM) is increasingly prevalent, contributing to a growing burden of cardiovascular disease (CVD) [1,2]. Patients with diabetes have a two- to four-fold higher risk of developing CVD compared to adults without diabetes [3,4]. As a result, cardiovascular risk management is a critical aspect of T2DM care, particularly in elderly patients [5]. Substantial evidence highlights the benefits of simultaneously addressing multiple risk factors to reduce cardiovascular complications and mortality in patients with diabetes [6–9]. This aligns with the current trend in treatment guidelines, which emphasize a multifactorial approach to cardiovascular risk management in T2DM patients [10]. Dyslipidemia, hypertension, and hyperglycemia are the three primary risk factors for CVD [11]. However, several studies indicate that not all risk factors contribute equally to reducing cardiovascular risk in diabetes patients [12,13]. Based on current evidence, lipid control appears to be the most critical factor when achieving targets for all three risk factors is not feasible. Therefore, optimizing lipid-lowering therapies in T2DM patients should be prioritized as a key strategy to reduce the overall burden of CVD [10,12].

Numerous clinical trials have demonstrated the benefits of statin therapy in the prevention of ASCVD. A meta-analysis, which included data from more than 18,000 people with diabetes from 14 randomized trials of statin therapy (mean follow-up of 4.3 years), showed that a 1 mmol/L (39 mg/dL) reduction in low-density lipoprotein cholesterol (LDL-C) resulted in a 21% reduction in the risk of CVD and a 9% reduction in all-cause mortality [14]. Therefore, statins are the preferred medication for lowering LDL-C and for reducing cardiovascular risk. The ADA 2023 guidelines for the diagnosis and treatment of type 2 diabetes mellitus recommend prescribing moderate-intensity statins for all diabetic patients aged 40–75, regardless of their 10-year atherosclerotic cardiovascular risk. For patients with high or very high cardiovascular risk, the guidelines recommend the use of high-intensity statins [10].

Despite strong recommendations from international and national guidelines [15–20], real-world practice in many Asian countries reveals a consistent gap between guideline-directed statin therapy and actual prescribing behavior, with high-intensity statins remaining underutilized. For example, a multicenter study in India reported that while more than 85% of patients received moderate-intensity statins, only 12.7% were prescribed high-intensity therapy [21]. These findings demonstrate a persistent challenge in translating evidence-based lipid management into daily practice.

This issue is especially relevant in type 2 diabetes (T2DM), a population with markedly elevated ASCVD risk, where intensive lipid-lowering therapy is often warranted. Yet, in Vietnam, data on real-world statin prescribing and treatment alignment with cardiovascular risk among outpatients with T2DM are lacking, particularly in settings caring for older patients with multiple comorbidities. Addressing this evidence gap, the present study was conducted at Friendship Hospital — a large public hospital managing a substantial number of elderly patients with T2DM — to evaluate statin prescribing patterns and the degree of concordance between statin intensity and cardiovascular risk. The results are expected to provide an evidence base for future quality improvement initiatives in lipid management for this high-risk population.

## Methods and materials

### Study subjects

The study included T2DM outpatients aged 40–75 at Friendship Hospital.

### Study methods

#### Study design.

A retrospective cohort study was conducted from electronic medical records covering the period from December 2022 to December 2023. Data were collected on demographic characteristics, medical history, cardiovascular risk factors, and cardiovascular risk target control using the hospital’s outpatient medical record system and patient interviews. The research team accessed the medical records for research purposes between January 2024 and March 2024. All data extracted from the hospital information system were fully anonymized prior to analysis, and the authors did not have access to information that could identify individual participants during or after data collection.

### Ethics approval

This retrospective study was conducted in accordance with the Declaration of Helsinki and approved by the Hospital Science and Technology Committee at Friendship Hospital. The requirement for written informed consent was waived by the Hospital Science and Technology Committee due to the retrospective nature of the study and the use of anonymized data. No identifiable personal information was included in the analysis or publication of this research.

### Sample size and sampling technique

#### Sample size.

The sample size was calculated using a single population proportion formula [22]. The study aimed to determine the proportion of patients prescribed statin therapy at an intensity appropriate to their cardiovascular risk (the key target in lipid management). Since no previous studies had reported this rate among diabetic patients at the study hospital, the research team assumed a proportion of 0.5 to estimate the maximum required sample size.


$$\text{n}=\frac{{\text{Z}}_{\left(\text{1}-\frac{\alpha }{\text{2}}\right)\;}^{\text{2}}\times \text{p}\times (\text{1}-\text{p})}{{\text{d}}^{\text{2}}}$$

where:

n: sample size.

Z: confidence level of 95% (Z = 1.96).

p is the estimated proportion of patients prescribed statin therapy at an intensity appropriate to their cardiovascular risk. Choose p = 0.5 to have the largest sample size.

d: Deviation between sample and population parameters, choose d = 0.05.

The calculated minimum sample size for the study was 385 patients.

The expected sample size for the study is 400 patients.

### Sample technique

Using the stratified random sampling method, with the treatment department as the stratification criterion, the list of diabetic outpatients in each department was randomly arranged using Excel 2016. Patients were then selected sequentially from the top of the list, based on the inclusion and exclusion criteria, until the required sample size for each department was achieved.

### Data collection

Patient data were retrospectively extracted from electronic medical records and included demographic information (age, sex, BMI, smoking status), clinical characteristics (diabetes duration, comorbidities such as hypertension, dyslipidemia, coronary artery disease, and chronic kidney disease), and laboratory results (LDL-C, HbA1c, ALT, renal function).

Statin prescription data were collected, including statin type, dosage, intensity classification (high, moderate, or low), and prescription frequency over the study period. The continuity of statin prescriptions was assessed based on the proportion of clinic visits with statin prescriptions. Additionally, LDL-C target achievement was evaluated according to ADA 2023 guidelines, and comparisons were made between patients with and without ASCVD.

### Definitions and evaluation criteria

#### Cardiovascular risk assessment of patients.

Cardiovascular risk stratification was conducted according to the 2023 American Diabetes Association (ADA) Standards of Care in Diabetes as follow:

- Patients with established ASCVD
- Patients without ASCVD:
  - High risk: ASCVD risk score ≥ 20%
  - Intermediate risk: 7.5% ≤ ASCVD risk score < 20%
  - Low risk: ASCVD risk score < 7.5%

In this study, ASCVD includes the following conditions: acute myocardial infarction (MI), acute coronary syndrome (ACS), coronary or other arterial revascularization, stroke, transient ischemic attack (TIA), and peripheral arterial disease (PAD).

### Assessment of LDL-C target achievement

The LDL-C target for each patient was determined according to the 2023 American Diabetes Association (ADA) Standards of Care in Diabetes, with specific criteria as follows:

- Patients with established ASCVD: LDL-C < 55 mg/dL (1.4 mmol/L).
- Patients without ASCVD, aged 40–75 years, with one or more atherosclerotic risk factors (obesity, hypertension, smoking, dyslipidemia, or chronic kidney disease/albuminuria): LDL-C < 70 mg/dL (1.8 mmol/L).
- Patients without ASCVD, aged 40–75 years, with no atherosclerotic risk factors: LDL-C < 100 mg/dL (2.6 mmol/L).

### Assessment of statin prescribing

Statin prescribing patterns were assessed to evaluate the appropriateness and consistency of statin therapy. The intensity of statin therapy (high, moderate, or low) was determined based on the statin type and dose prescribed at the most recent outpatient visit during the study period. This time point was used to represent the current prescribing practice.

In addition, the continuity of statin prescribing was assessed over a 12-month period (from December 5, 2022 to December 5, 2023). Continuity was defined as the proportion of total clinic visits during which a statin was prescribed for each patient and categorized as:

- 100% of visits (fully continuous)
- 50% to <100% of visits (partially continuous)
- < 50% of visits (poor continuity)
- No statin prescribed during the entire year

Changes in statin intensity during the follow-up period were also documented. During the one-year follow-up period, a change in statin therapy was defined as any modification to the statin regimen, including initiation or discontinuation of statin treatment, switching between statin agents, change in statin dose resulting in a different intensity category. Any of these events occurring at least once during follow-up was classified as a treatment change.

### Statin prescribing criteria

We evaluated prescription appropriateness based on the ADA Standards of Care 2023, Section 10.

Secondary prevention (clinical ASCVD, any age): high-intensity statin (or maximally tolerated dose), add-on therapy (e.g., ezetimibe/PCSK9i) is recommended if the goal is not achieved.

Primary prevention (age 40–75 years with diabetes): at least moderate-intensity statin for all. For those at higher cardiovascular risk with ≥1 ADA-listed ASCVD risk factor (diabetes duration, overweight/obesity, hypertension, dyslipidemia, smoking, family history of premature CAD, chronic kidney disease, or albuminuria) and/or elevated 10-year ASCVD risk—high-intensity statin is recommended.

### Statistical analysis

Data was entered and analyzed using R software version 4.2.1. Continuous variables were expressed as the mean ± standard deviation (SD) if they followed a normal distribution or as the median and interquartile range (IQR) if they did not. Nominal and categorical variables were presented as frequencies and percentages. To test differences between two independent groups: (1) For continuous variables, a T-test was used if the data were normally distributed, and the Mann-Whitney U test was applied if they were not. (2) For nominal variables, the Chi-square test or Fisher’s exact test was used as appropriate. Results were considered statistically significant if p < 0.05.

Multivariate logistic regression was conducted to identify factors associated with whether or not patients were prescribed statins in the study population. The Backward Stepwise (Wald) method was applied, using a p-value threshold of 0.10 for variable inclusion or removal.

Independent variables were selected based on previous research findings and clinical relevance, including demographic characteristics, cardiovascular risk factors, and laboratory parameters. These variables were first examined through univariate analysis to determine their eligibility for inclusion in the logistic regression model.

The results of the regression analysis were reported as odds ratios (ORs) with 95% confidence intervals (CIs). A p-value < 0.05 was considered statistically significant in determining factors associated with statin prescription patterns.

## Results

### Demographics and characteristics of study participants

**Fig 1:**
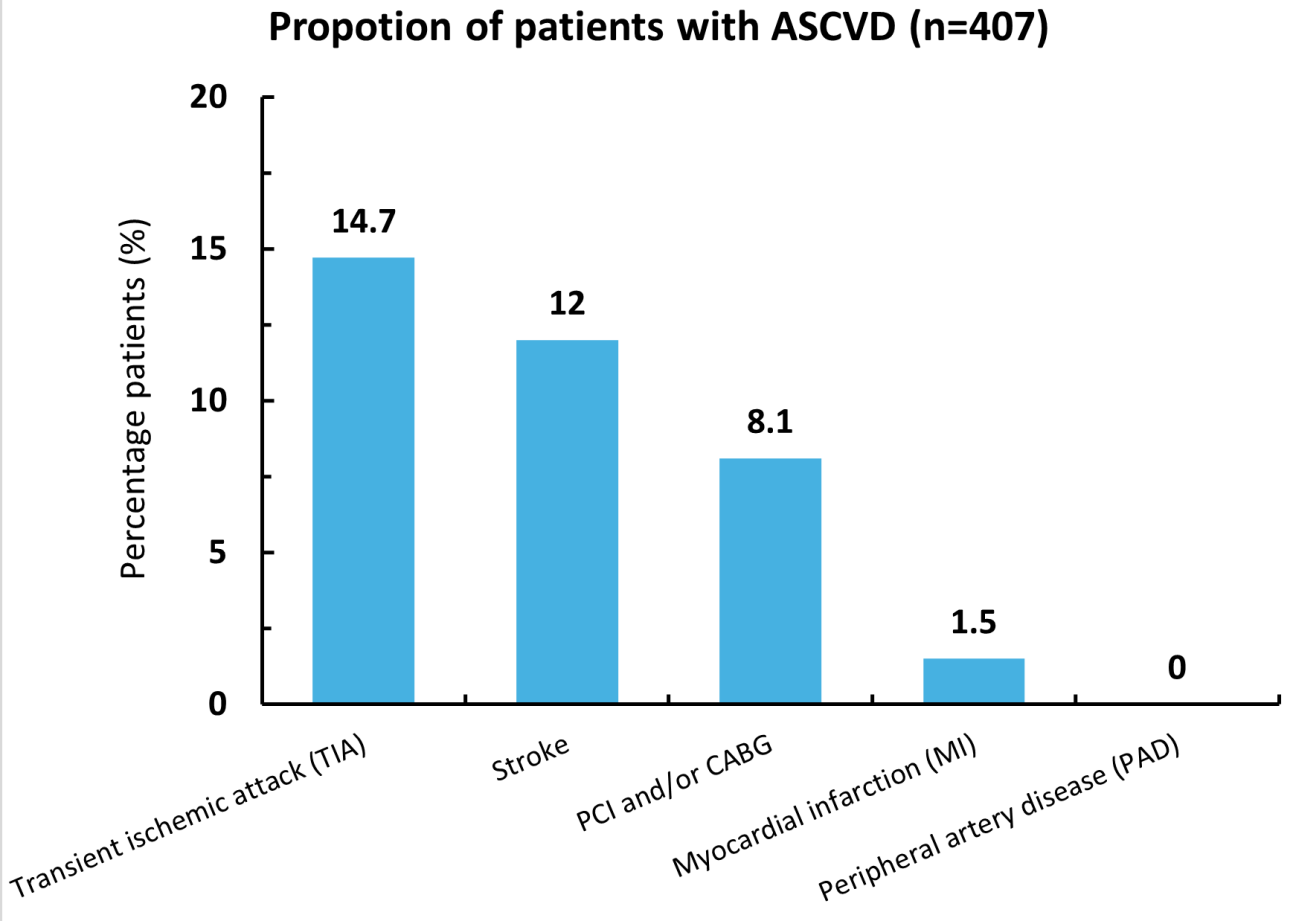
Proportions of patients with ASCVD. ASCVD: Atherosclerotic cardiovascular disease; CABG: Coronary artery bypass grafting; PCI: Percutaneous coronary intervention; PAD: Peripheral arterimeal disease; TIA: Transient ischemic attack.

The demographic characteristics of the study participants are presented in Table 1 and Fig 1. A total of 407 patients aged 40–75 years were included, with a median age of 70 years (IQR: 66–73); 99.6% were aged ≥50 years. The male-to-female ratio was 2.9, and 57.0% of patients were classified as overweight or obese (BMI ≥ 23 kg/m^2^). No significant differences were found between patients with and without ASCVD in terms of age, sex, smoking status, or BMI (p > 0.05).

**Table 1:** Characteristics of the study population (N = 407).

Characteristics	Frequency n (%) (N = 407)	ASCVD condition		p-value
		Established ASCVD n (%) (N_1_ = 124)	Non – ASCVD n (%) (N_2_ = 283)	
***Gender***				
Male	303 (74.4)	92 (74.2)	211 (74.6)	0.974
***Age group***				0.163
40–49 years old	2 (0.5)	0 (0)	2 (0.7)	
50–69 years old	185 (45.5)	49 (39.5)	136 (48.1)	
70–75 years old	220 (54.1)	75 (60.5)	145 (51.2)	
***Smoking***	74 (18.2)	22 (17.7)	52 (18.4)	
***BMI* (*kg/m***^***2***^)	*23.4 (21.95–25)* ^*1*^			0.725
BMI < 23	175 (43.0)	57 (46.0)	118 (41.7)	
23 ≤ BMI < 25	128 (31.4)	37 (29.8)	91 (32.2)	
BMI ≥ 25	104 (25.6)	30 (24.2)	74 (26.1)	
***Treatment department***				0.011
Medical examination B	206 (50.6)	60 (48.4)	146 (51.6)	
Cardiology	30 (7.4)	17 (13.7)	13 (4.6)	
Internal A	61 (15.0)	19 (15.3)	42 (14.8)	
Endocrinology – Diabetes	110 (27.0)	28 (22.6)	82 (29.0)	
***Duration of diabetes type 2 (years)***	*10 (5–17)* ^*1*^			*0.408*
< 10 years	165 (40.5)	46 (37.1)	119 (42.0)	
≥ 10 years	242 (59.5)	78 (62.9)	164 (58.0)	
***eGFR (ml/min/1.73 m*** ^***2***^ **)**	*67 (57.7–77.2)* ^*1*^			
eGFR < 30	3 (0.7)	2 (1.6)	1 (0.4)	0.166
30 ≤ eGFR < 45	18 (4.4)	5 (4.0)	13 (4.6)	
45 ≤ eGFR < 60	102 (25.1)	38 (30.6)	64 (22.6)	
eGFR ≥ 60	284 (69.8)	79 (63.7)	205 (72.4)	
***Comorbidities***				
Coronary artery disease (CAD)	213 (52.3)	88 (71.0)	125 (44.2)	**0.013**
Hypertension	313 (76.9)	102 (82.3)	211 (74.6)	0.539
Dyslipidemia	398 (97.8)	123 (99.2)	275 (97.2)	0.887
Chronic kidney disease (CKD)	162 (39.8)	61 (49.2)	101 (35.7)	0.143
Heart failure	35 (8.6)	18 (14.5)	17 (6.0)	0.06
***Eligible for high-intensity statin***	406 (99.8)	124 (100.0)	282 (99.6)	–

**ASCVD**: Atherosclerotic cardiovascular disease; **BMI**: Body mass index; **CABG**: Coronary artery bypass grafting; **eGFR**: Estimated glomerular filtration rate; **PCI**: Percutaneous coronary intervention; **T2DM**: Type 2 diabetes mellitus; **ULN**: Upper limit of normal; **p-value**: The p-value from the Chi-square test comparing each characteristic between patients with and without ASCVD; **Median** (interquartile range – IQR)

A diabetes duration of ≥10 years was observed in 59.5% of patients. The most common comorbidities were dyslipidemia (97.8%), hypertension (76.9%), and coronary artery disease (52.3%), followed by CKD (39.8%). ALT levels were within the normal range in 84.8% of patients.

ASCVD was identified in 30.5% of patients. Among these, transient ischemic attack (14.7%) and stroke (12.0%) were the most frequent manifestations. Myocardial infarction was reported in 1.5% of patients, and no cases of peripheral artery disease were recorded. Additionally, 8.1% of patients had a history of percutaneous coronary intervention or coronary artery bypass grafting.

### Characteristics of patients’ cardiovascular risk stratification and LDL-C target achievement rate

**Fig 2:**
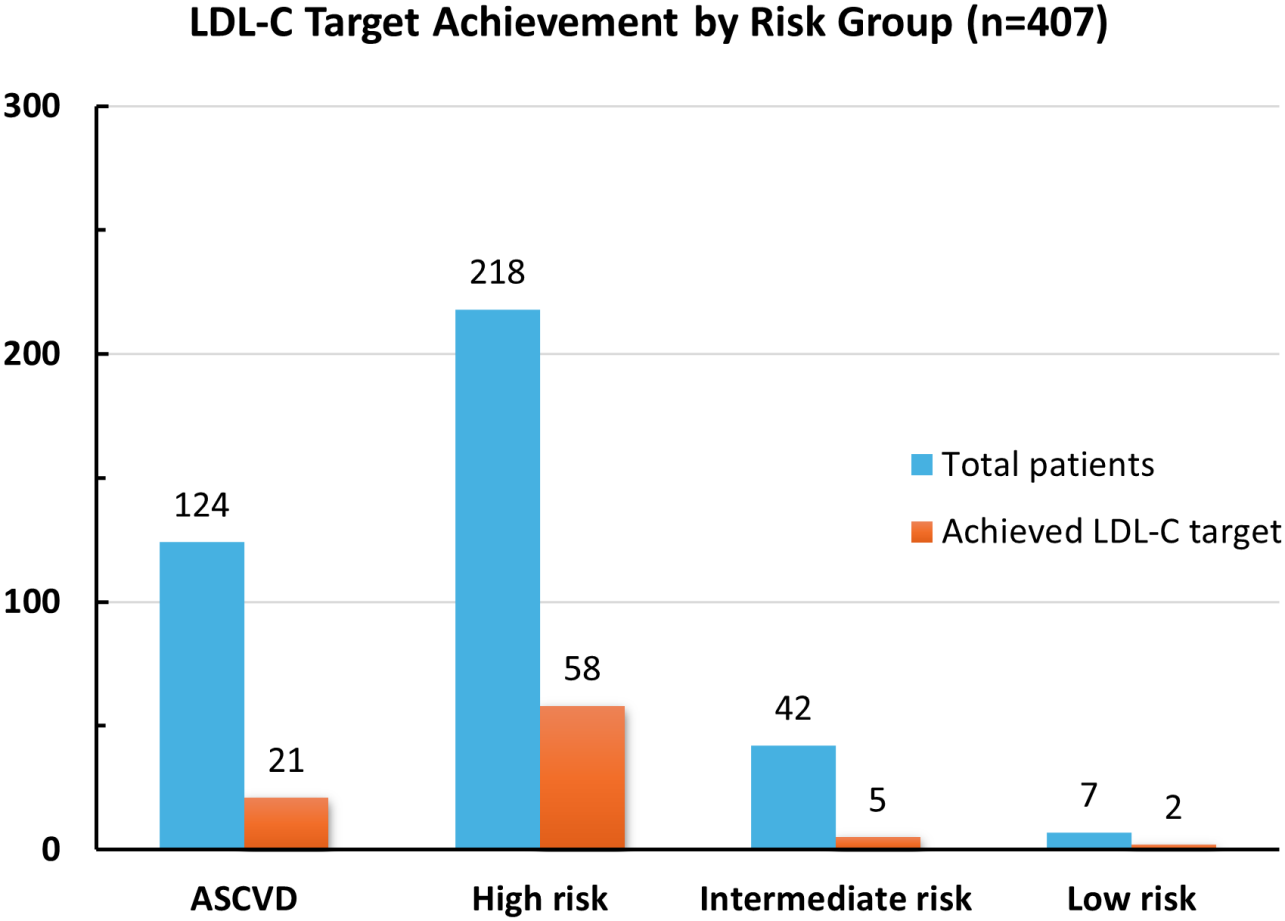
Cardiovascular risk characteristics andLDL-C target achievement rates of patients.

Characteristics of patients’ cardiovascular risk stratification and LDL-C target achievement rate are presented in Fig 2. Among patients without ASCVD, 218 had high cardiovascular risk, 42 had intermediate risk, 7 had low risk, and 16 could not be classified using the ASCVD Risk Estimator Plus. The LDL-C target achievement rates, according to the 2023 ADA Guidelines, were 26.6% in the high-risk group, 11.9% in the intermediate-risk group, and 28.6% in the low-risk group. Among patients with established ASCVD, 16.9% achieved the LDL-C target.

### Characteristics of statin prescriptions

Key characteristics of statin prescribing in the study population are presented in Table 2.

**Table 2:** Statin prescription characteristics by LDL-C target achievement status.

Characteristics	Frequency n (%) (N = 407)	Achieving LDL – C target		p-value^*^
		Achieving n(%)	Not achieving n(%)	
**Intensity of statin**				**0.30**
High-intensity	0 (0.0)	0 (0.0)	0 (0.0)	–
Moderate-intensity + ezetimibe	44 (10.8)	8 (18.2)	36 (81.8)	
Moderate-intensity	271(66.6)	52 (19.2)	219 (80.8)	
Low-intensity	35 (8.6)	7 (20.0)	28 (80.0)	
Not prescribed statin	57 (14.0)	5 (8.8)	52 (91.2)	
**Continuity of statin prescribing over 1 year**				0.13
< 50% of total visits	48 (11.8)	4 (8.3)	44 (91.7)	
50% to < 100% of total visits	121 (29.7)	26 (21.5)	95 (78.5)	
100% of total visits	238 (58.5)	42 (17.6)	196 (82.4)	
**Change in statin intensity over 1 year**				0.35
Yes	221 (54.3)	35 (15.8)	186 (84.2)	
No	186 (45.7)	37 (19.9)	149 (80.1)	

* Chi – square test.

**Fig 3:**
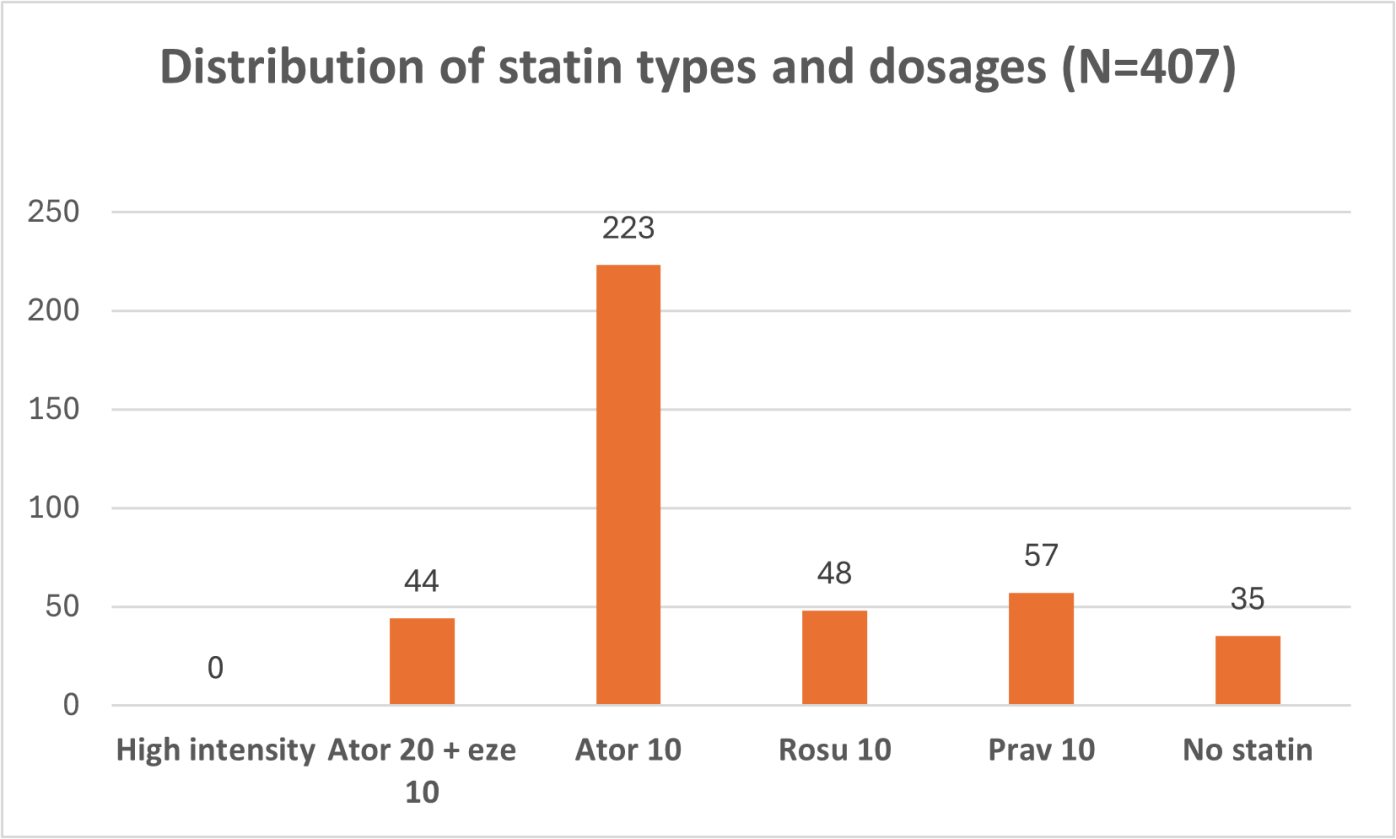
Distribution of statin types and dosages. Ator: atorvastatin, Eze: ezetimibe, Rosu: rosuvastatin, Prav: pravastatin.

**Fig 4:**
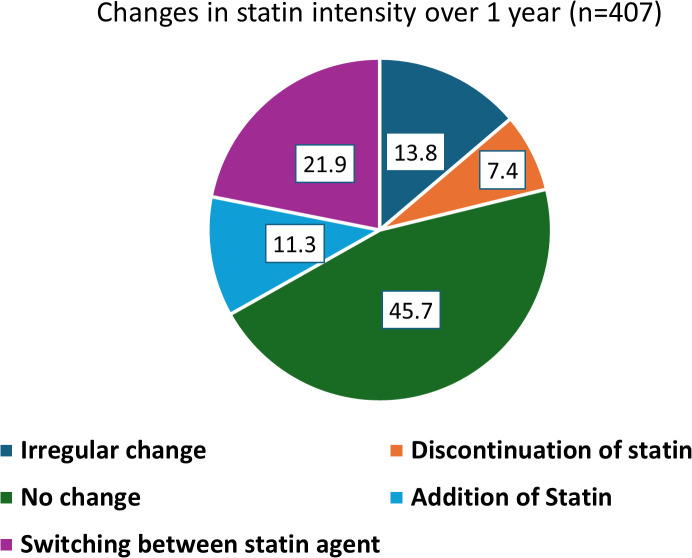
Changes in the statin intensity over a year.

Among the 406 patients with an indication for high-intensity statin therapy, none were prescribed high-intensity statins. Instead, 77.4% received moderate-intensity statins, 8.6% received low-intensity statins, and 14.0% were not prescribed any statin. Over the 12-month observation period, 58.5% of patients were prescribed statins at every clinic visit. Additionally, changes in statin intensity were observed in 54.3% of patients. The detailed distribution of statin types and dosages prescribed, as well as the specific patterns of changes in statin therapy are presented in Figs 3 and 4, respectively.

There was no significant association between statin intensity and LDL-C target achievement (χ²(3)=3.67, p = 0.30). Similarly, LDL-C target achievement did not differ significantly by the continuity of statin prescribing (χ²(2)=4.08, p = 0.13) and the change in statin intentsity (χ²(1)=0.88, p = 0.35).

### Factors influencing statins prescription

Factors associated with whether or not patients were prescribed statins in the study population are summarized in Table 3. Multivariate logistic regression analysis identified dyslipidemia, established ASCVD, and treatment department as significant predictors of statin use. Patients with established ASCVD were 1.85 times more likely to be prescribed statins compared to those without ASCVD (adjusted OR: 1.85; p = 0.05). Dyslipidemia showed a particularly strong association, with affected patients being 15.78 times more likely to receive statin therapy (p < 0.001). Additionally, compared to patients managed in the Medical Examination B department, those treated in the Endocrinology and Cardiology departments were significantly more likely to be prescribed statins, with adjusted odds ratios of 7.90 and 7.65, respectively.

**Table 3:** Factors influencing statins prescription by multivariate analysis.

Factors	Univariate OR (95% CI)	p-value	Multivariate OR (95% CI)	p-value
**Female**	0.97 (0.55–1.71)	0.914		
**Age > 70**	0.8 (0.49–1.33)	0.4648		
**BMI (ref: < 23)**				
*23–25*	0.86 (0.49–1.51)	0.888		
*>25*	1.43 (0.74–2.78)	0.251		
**Smoking**	0.81 (0.44–1.51)	0.6226		
**Treatment department** **(ref: Examination B)**		**< 0.0001**		
*Internal A*	1.12 (0.58–2.16)	0.975	1.06 (0.53–2.1)	0.874
*Endocrine*	7.65 (2.96–19.75)	<0.001	7.9 (2.97–21.04)	<0.001
*Cardiology*	5.1 (1.18–22.12)	0.014	7.65 (1.41–41.42)	0.018
**Duration of diabetes ≥ 10 years** **(ref: < 10 years)**	1.05 (0.64–1.74)	0.9417		
**Very high-risk**	1.71 (0.87–3.34)	0.165		
**ASCVD**	1.85 (1.02–3.37)	0.0556	**1.85 (0.98–3.51)**	**0.05**
**High blood pressure**	1.09 (0.61–1.95)	0.8747		
**Dyslipidemia**	16.4 (3.34–80.82)	**< 0.0001**	15.78 (2.75–90.62)	**< 0.001**
**Chronic kidney failure**	0.91 (0.55–1.51)	0.8258		
**Heart failure**	0,77 (0.33–1.76)	0.6917	0.4 (0.15–1.09)	0.081
**Achieving blood pressure target**	0.77 (0.43–1.37)	0.446		
**Achieving LDL-C target**	2.45 (1.08–5.59)	0.0423	1.81 (0.77–4.26)	0.153
**Achieving HbA1c target**	1.15 (0.66–2)	0.7179		

*Note: OR: odds ratio, BMI: body mass index, ASCVD: atherosclerotic cardiovascular disease, ref: reference.*

## Discussion

This study provides an in-depth assessment of real-world statin prescribing pattern among outpatients with type 2 diabetes mellitus (T2DM) at a major public hospital in Vietnam. The findings highlight key issues related to suboptimal statin use, particularly in terms of treatment intensity and alignment with cardiovascular risk.

To date, there has been a lack of published data in Vietnam specifically evaluating the appropriateness of statin prescribing in patients with T2DM. This study is among the first to apply ADA guideline criteria to assess real-world prescribing quality in this high-risk population in Vietnam, providing essential baseline evidence to inform future quality improvement efforts.

In this study, the majority of patients were older adults with long-standing type 2 diabetes and multiple comorbidities, placing them at elevated cardiovascular risk. Based on the 2023 ADA guidelines, all individuals with established ASCVD or with one or more cardiovascular risk factors should be considered at higher risk and may benefit from high-intensity statin therapy. Among the 407 patients included, 406 (99.8%) met the criteria for high-intensity statin therapy, either due to established ASCVD or the presence of additional risk factors such as hypertension, dyslipidemia, obesity, or chronic kidney disease. Despite this, none of the eligible patients were prescribed high-intensity statins, and the overall LDL-C target achievement rate was only 17.7%. Notably, the proportion of patients achieving LDL-C goals remained consistently low across all cardiovascular risk strata, including those with established ASCVD (16.9%), high-risk individuals without ASCVD (26.6%), and those with intermediate or low risk (11.9% and 28.6%, respectively). These findings indicate that suboptimal lipid management was not restricted to a specific risk group, but rather reflected a widespread gap in the application of guideline-directed therapy in routine outpatient care. Bridging this gap is critical to reducing residual cardiovascular risk in patients with diabetes.

While the statin prescription rate in our population was relatively high (86.0%), the low proportion of patients achieving LDL-C targets reflects a mismatch between prescribed intensity and cardiovascular risk. Among the 406 patients with an indication for high-intensity statin therapy, none were prescribed high-intensity statins. No usage of high-intensity statins may be related to concerns about adverse effects, underestimation of cardiovascular risk, reluctance to initiate high-dose therapy, and limited availability of some high-dose statin options. Although rosuvastatin 20 mg, a high intensity statin, is available in the study hospital, there was no prescription at this dosage at all. Further research in Vietnam is needed to clarify these factors. Moreover, 8.6% of patients received low-intensity statins—an intensity generally not recommended in patients with T2DM unless higher doses are not tolerated—and 14.0% were not prescribed any statins at all. Furthermore, more than half of the patients had changes in statin intensity during follow-up. The observed treatment modifications encompassed a range of regimen changes—not only escalation or reduction in statin intensity but also switching agents, adding or discontinuing lipid-lowering therapy, and treatment interruptions. Given this heterogeneity, the high frequency of changes likely reflects instability in lipid-lowering management rather than consistent therapy optimization. These patterns raise concerns about potential barriers to optimal prescribing, including lack of cardiovascular risk stratification, limited awareness of guideline updates, or concerns about tolerability and safety of high-intensity regimens.

To contextualize our findings within the regional landscape, evidence from multiple Asian countries consistently demonstrates that suboptimal statin prescribing in patients with type 2 diabetes is a pervasive problem. In China, a population-based study of more than 700,000 patients revealed that only 31.5% were prescribed statins, with rates dropping to 15% in those without established cardiovascular disease, and high-intensity regimens remained exceedingly rare [23]. Similarly, data from Thailand showed that fewer than 9% of diabetic patients achieved high-intensity statin use, while the majority were maintained on moderate- or low-intensity therapy despite being at very high cardiovascular risk [24]. Findings from India further highlighted that just over half of patients received any statin therapy, and only 12.7% were on high-intensity regimens [21]. Even in Malaysia, where overall prescription rates were relatively high (above 80%), the majority of patients received simvastatin at moderate or low doses, and less than 40% attained LDL-C treatment goals [25,26]. Taken together, the evidence highlights a substantial discrepancy between clinical guideline recommendations and actual prescribing practices in Asia. Although the overall use of statins has gradually increased, the adoption of high-intensity regimens remains uncommon, resulting in persistently low rates of LDL-C target achievement. This pattern is not unique to one country but reflects a broader regional challenge, where underutilization of intensive therapy limit cardiovascular risk reduction in patients with diabetes. Our findings are consistent with this regional experience and further reinforce the need for stronger efforts to optimize statin prescribing in order to close the gap between evidence-based recommendations and real-world outcomes.

The low rate of LDL-C target achievement in this study may also reflect limitations in therapeutic decision-making, or limited availability of combination lipid-lowering therapy. While most patients had normal liver function and were eligible for statin use, clinical inertia or misconceptions about safety may have hindered the use of high-intensity therapy. Moreover, fragmented care across departments was found to significantly influence prescribing patterns, suggesting that greater multidisciplinary collaboration may be needed to standardize risk-based lipid management approaches.

Importantly, this study addresses a gap in the Vietnamese literature by providing one of the first comprehensive analyses of statin prescribing patterns in T2DM outpatients in a real-world hospital setting. By highlighting the disconnect between cardiovascular risk and treatment intensity, it underscores an urgent need for interventions to improve adherence to guideline-directed statin therapy, including clinician education, decision support tools, and audit-feedback mechanisms.

Our findings regarding factors associated with statin prescribing are consistent with prior research conducted in Asian populations. Similar to our results, study from Malaysia also identified dyslipidemia as the strongest predictor of statin use, reflecting physicians’ greater tendency to initiate lipid-lowering therapy when abnormal laboratory results are present rather than solely on the basis of cardiovascular risk stratification [26]. The influence of treatment department is noteworthy, suggesting that specialist care in Endocrinology and Cardiology departments is more strongly aligned with guideline-based practice than general outpatient clinics. In our hospital, Examination B is a general outpatient clinic where internists manage a wide range of medical conditions. Because diabetes care is not the primary clinical focus in this setting, statin prescribing may be less emphasized compared with specialized cardiology or endocrine outpatient clinics that routinely implement guideline-directed lipid management. This trend also was reported in Indian and Thai studies, where referral to specialized clinics was associated with higher statin utilization and more appropriate dosing [21,24]. From a practical perspective, these results underscore the importance of strengthening risk-based prescribing across all care settings, not only in specialty clinics. Integrating systematic cardiovascular risk assessment tools, reinforcing continuing medical education, and embedding clinical decision support into electronic health records may help mitigate therapeutic inertia and reduce disparities in statin use between departments. Such measures are critical to ensure that all high-risk patients, regardless of care setting, receive appropriate lipid-lowering therapy in line with guideline recommendations.

### Limitations

This study has several limitations that should be considered when interpreting the findings. First, the single-center design restricts causal inference and may limit the generalizability of the results. However, the hospital involved is a large tertiary facility that manages a high volume of patients with type 2 diabetes and cardiovascular comorbidities, making the sample representative of typical outpatient populations in similar urban public hospitals in Vietnam.

Second, data were collected retrospectively from electronic medical records, which may have resulted in missing or incomplete documentation, particularly regarding the rationale for prescribing decisions or reasons for therapy discontinuation. Nevertheless, the study focused on objective indicators such as statin type, intensity, and laboratory values, which are routinely recorded and reliably captured in the hospital information system.

Third, the assessment of statin treatment continuity was based on prescription records rather than direct measures of patient adherence. While this may not reflect actual medication use, prescription patterns still offer valuable insight into clinician behavior and the consistency of statin therapy across follow-up visits—an important dimension of real-world practice.

Fourth, the study did not assess non-clinical factors such as medication availability, insurance coverage, or patients’ financial capacity, all of which may influence prescribing behavior. However, since statins are widely available and covered by national health insurance in Vietnam, and the study was conducted in a setting where patients typically have access to standard therapies, this limitation is unlikely to have significantly biased the findings.

Despite these limitations, the study provides important real-world evidence on the disconnect between cardiovascular risk and statin prescribing intensity, underscoring the need for system-level interventions to promote adherence to guideline-directed lipid-lowering therapy in patients with type 2 diabetes.

## Conclusion

Statins are available, cost-effective, and safe, with well-established cardiovascular benefits. However, challenges persist in clinical practice, particularly in determining the appropriate statin intensity for patients at varying levels of cardiovascular risk. To improve the quality of statin prescribing, it is essential to enhance education, foster multidisciplinary collaboration in setting LDL-C targets, and ensure consistent treatment monitoring.

## Supporting information

S1 File Statin agents available at the hospital. (DOCX)

## Abbreviations

ACC: The American College of Cardiology
ACS: Acute Coronary Syndrome
ADA: American Diabetes Association
AHA: The American Heart Association
ALT: Alanine aminotransferase
BMI: Body Mass Index
CAD: Coronary Artery Disease
CABG: Coronary artery bypass grafting
CCS: Chronic Coronary Syndromes
CIs: Confidence Intervals
CKD: Chronic Kidney Disease
CK: Creatine kinase
CVD: Cardiovascular disease
eGFR: Estimated glomerular filtration rate
IQR: Interquartile range
LDL-C: Low-Density Lipoprotein – Cholesterol
OR: Odd Ratio
PAD: Peripheral arterial disease
PCI: Percutaneous coronary intervention
RCT: Randomized Controlled Trials
SD: Standard deviation
T2DM: Type 2 Diabetes Mellitus
TIA: Transient ischemic attack
ULN: Upper limit of normal
