# Supplementary material for: Statin prescribing patterns in cardiovascular risk management among outpatients with type 2 diabetes: Real-world practices at a Vietnamese general hospital
Source: PLoS One. 2026 Feb 27;21(2):e0343313. doi: 10.1371/journal.pone.0343313 (PMC12948102; doi:10.1371/journal.pone.0343313)
Supplement: S1 File — (DOCX) [file pone.0343313.s001.docx]

**Statin agents available at the hospital during the study period**

**Monotherapy formulations:**

- ***Low-intensity statin:***
  Pravastatin 10 mg
- ***Moderate-intensity statins:***
  Atorvastatin 10 mg
  Atorvastatin 20 mg
  Rosuvastatin 5 mg
  Rosuvastatin 10 mg
- ***High-intensity statin:***
  Rosuvastatin 20 mg

**Fixed-dose combination formulations:**

- Atorvastatin 10 mg + Amlodipine 5 mg
- Atorvastatin 20 mg + Amlodipine 5 mg
- Atorvastatin 20 mg + Ezetimibe 10 mg
